# Mobile Air Quality Studies (MAQS)-an international project

**DOI:** 10.1186/1745-6673-5-8

**Published:** 2010-04-09

**Authors:** David A Groneberg, Cristian Scutaru, Mathias Lauks, Masaya Takemura, Tanja C Fischer, Silvana Kölzow, Anke van Mark, Stefanie Uibel, Ulrich Wagner, Karin Vitzthum, Fabian Beck, Stefanie Mache, Carolin Kreiter, Bianca Kusma, Annika Friedebold, Hanna Zell, Alexander Gerber, Johanna Bock, Khaled Al-Mutawakl, Johannes Donat, Maria Victoria Geier, Carolin Pilzner, Pia Welker, Ricarda Joachim, Harald Bias, Michael Götting, Mohannad Sakr, Johann P Addicks, Julia-Annik Börger, Anna-Maria Jensen, Sonja Grajewski, Awfa Shami, Niko Neye, Stefan Kröger, Sarah Hoffmann, Lisa Kloss, Sebastian Mayer, Clemens Puk, Ulrich Henkel, Robert Rospino, Ute Schilling, Evelyn Krieger, Gesa Westphal, Andreas Meyer-Falcke, Hagen Hupperts, Andrés de Roux, Salome Tropp, Marco Weiland, Janette Mühlbach, Johannes Steinberg, Anne Szerwinski, Sepiede Falahkohan, Claudia Sudik, Anna Bircks, Oliver Noga, Nicolas Dickgreber, Q Thai Dinh, Heiko Golpon, Beatrix Kloft, Rafael Neill B Groneberg, Christian Witt, Sabine Wicker, Li Zhang, Jochen Springer, Birgitta Kütting, Ervin C Mingomataj, Axel Fischer, Norman Schöffel, Volker Unger, David Quarcoo

**Affiliations:** 1 Department of Environmental and Traffic Medicine, The Institute of Occupational Medicine, Charité - Universitätsmedizin Berlin, Medical School of the Freie University Berlin and the Humboldt-University Berlin, Berlin, Germany; 2 Department of Informatics, The Institute of Occupational Medicine, Charité - Universitätsmedizin Berlin, Medical School of the Freie University Berlin and the Humboldt-University Berlin, Berlin, Germany; 3 Fachhochschule Senftenberg, Senftenberg, Germany; 4 Respiratory Disease Center, Kitano Hospital, Osaka, Japan; 5 Laser Centre, Potsdam, Germany; 6 Department of Allergy, The Institute of Occupational Medicine, Charité - Universitätsmedizin Berlin, Medical School of the Freie University Berlin and the Humboldt-University Berlin, Berlin, Germany; 7 Institute of Occupational Medicine, University of Lübeck, Lübeck, Germany; 8 Department of Toxicology, The Institute of Occupational Medicine, Charité - Universitätsmedizin Berlin, Medical School of the Freie University Berlin and the Humboldt-University Berlin, Berlin, Germany; 9 Chest Hospital Löwenstein, Löwenstein, Germany; 10 Department of Sports Medicine, The Institute of Occupational Medicine, Charité - Universitätsmedizin Berlin, Medical School of the Freie University Berlin and the Humboldt-University Berlin, Berlin, Germany; 11 Pariser Street Outpatient Clincis, Berlin, Germany; 12 Department of Health Management, The Institute of Occupational Medicine, Charité - Universitätsmedizin Berlin, Medical School of the Freie University Berlin and the Humboldt-University Berlin, Berlin, Germany; 13 Chest Department Heckeshorn, Helios-Emil-von-Behring-Hospital, Berlin, Germany; 14 Department of Occupational Psychology, The Institute of Occupational Medicine, Charité - Universitätsmedizin Berlin, Medical School of the Freie University Berlin and the Humboldt-University Berlin, Berlin, Germany; 15 Department of Surgery, Helios-Emil-von-Behring-Hospital, Berlin, Germany; 16 Rheumaklinik Berlin-Buch, Berlin, Germany; 17 Faculty of Medicine, University of Sanaa, Yemen; 18 Ruppiner Kliniken, Neuruppin, Germany; 19 Department of Respiratory Medicine, Centre of Medicine, Medizinische Hochschule Hannover, Hannover, Germany; 20 Department of Cell Biology, Mivenion Inc., Berlin, Germany; 21 AMZ, Charité - Universitätsmedizin Berlin, Berlin, Germany; 22 Al-Assaf University Hospital, Lattakia, Syria; 23 General Hospital, Freising, Germany; 24 Department of Oral and Maxillofacial Surgery, Charité - Universitätsmedizin Berlin, Medical School of the Freie University Berlin and the Humboldt-University Berlin, Berlin, Germany; 25 Faculty of Medicine, Tishreen University, Lattakia, Syria; 26 Department of Medicine, Park-Klinik Weissensee, Berlin, Germany; 27 Department of Neurology, Charité - Universitätsmedizin Berlin, Medical School of the Freie University Berlin and the Humboldt-University Berlin, Berlin, Germany; 28 Department of Surgery, Dominikus-Hospital, Berlin, Germany; 29 Occupational Medicine, TUV, Berlin, Germany; 30 Strategy Centre for Health, Health Care Campus North Rhine Westphalia, Bochum, Germany; 31 Chest Clinics Charlottenburg, Berlin, Germany; 32 Unfallkrankenkaus Marzahn, Berlin, Germany; 33 Hospital Luckenwalde, Luckenwalde, Germany; 34 Institute for Allergy and Asthma Research, Berlin, Germany; 35 Otto-Heubner-Centre, Charité - Universitätsmedizin Berlin, Medical School of the Freie University Berlin and the Humboldt-University Berlin, Berlin, Germany; 36 Faculty of Biology, University of Mainz, Mainz, Germany; 37 Department of Medicine, Charité - Universitätsmedizin Berlin, Medical School of the Freie University Berlin and the Humboldt-University Berlin, Berlin, Germany; 38 Occupational Medicine Service, University of Frankfurt, Frankfurt, Germany; 39 Fujian First College of Medicine, Fujian, PR China; 40 Division of Applied Cachexia Research and Center for Cardiovascular Research, Charité-Universitätsmedizin Berlin, Medical School of the Freie University Berlin and the Humboldt-University Berlin, Berlin, Germany; 41 Institute and Outpatient Clinic of Occupational, Social and Environmental Medicine, University of Erlangen- Nuremberg, Erlangen, Germany; 42 Dept of Allergology & Clinical Immunology, Mother Theresa School of Medicine, Tirana, Albania

## Abstract

Due to an increasing awareness of the potential hazardousness of air pollutants, new laws, rules and guidelines have recently been implemented globally. In this respect, numerous studies have addressed traffic-related exposure to particulate matter using stationary technology so far. By contrast, only few studies used the advanced technology of mobile exposure analysis. The Mobile Air Quality Study (MAQS) addresses the issue of air pollutant exposure by combining advanced high-granularity spatial-temporal analysis with vehicle-mounted, person-mounted and roadside sensors. The MAQS-platform will be used by international collaborators in order 1) to assess air pollutant exposure in relation to road structure, 2) to assess air pollutant exposure in relation to traffic density, 3) to assess air pollutant exposure in relation to weather conditions, 4) to compare exposure within vehicles between front and back seat (children) positions, and 5) to evaluate "traffic zone"-exposure in relation to non-"traffic zone"-exposure.

Primarily, the MAQS-platform will focus on particulate matter. With the establishment of advanced mobile analysis tools, it is planed to extend the analysis to other pollutants including NO2, SO2, nanoparticles and ozone.

## Introduction

Air pollution is one of the major global problems [1]. It can be defined as the emission of pollutants into the atmosphere by natural or anthropogenic sources and displays one of the main issues in environmental medicine [2-4]. Anthropogenic air pollution commenced with human's systematic use of fire thousands of years ago. Today the major sources of anthropogenic air pollution are factory emissions, the burning of fuels and street traffic. In the later, especially exhaust gases and tire abrasion are a major problem. Currently, there is a major debate on the impact of these traffic-related pollutants on local air quality in the urban and also rural environment. Since polluted air can deteriorate conditions such as asthma, COPD or increase cardiovascular risks [1], most countries have strengthened laws to control the air quality in the past decade. Polluted air is considered as a super-regional problem. Therefore, international conferences have recently developed different ways to improve and assure air quality employing global strategic perspectives.

In striking contrast to the amount of research that is currently conducted in the field of health effects [1], only little is known on specific exposure situations.

Whereas there is a large amount of data available using stationary systems, only little is known about the practicability of mobile sensors in the assessment of air pollution.

To address this issue the authors of this protocol have decided to establish a platform for Mobile Air Quality Studies (MAQS). The present article describes the background and study protocol of this international project.

**Figure 1:**
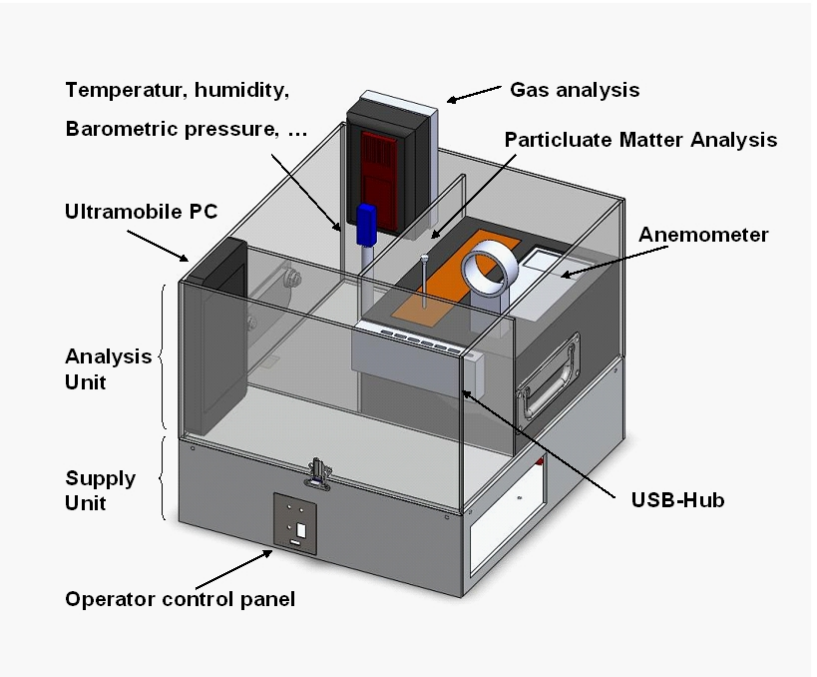
**MAQS-vehicle sensing modules**. Varible positions of sensors with regard to positions within the vehicle (front or back seats) and sensor position height (adults or children height).

As primary mobile technology platform for MAQS convertible vehicles were chosen. They offer the advantage of assessing air quality in both static and mobile modes. Within the vehicles, different positions of the sensing modules can be also selected to monitor driver and co-driver exposure under different settings (Fig. 1). Secondarily, bicycle, motor bicycle and pedestrian solutions will be developed.

## Aims

1. To assess air pollutant exposure in relation to urban and rural infrastructure,

2. To assess air pollutant exposure in relation to road structure,

3. To assess air pollutant exposure in relation to traffic density,

4. To assess air pollutant exposure in relation to weather conditions and other outdoor air quality parameters,

5. To assess air pollutant exposure in relation to vehicle air ventilation and air condition (different settings),

6. To assess CO_2 _values in relation to particulate matter exposure,

7. To compare exposure between front and back seat (children) positions

8. To evaluate "traffic zone"-exposure in relation to non-"traffic zone"-exposure

9. To generate recommendations concerning the use of the open vehicle position in relation to road structure

10. To generate recommendations concerning the use of the open vehicle position in relation to traffic density with special regard to traffic congestion.

## Methods

### Monitoring

**Figure 2:**
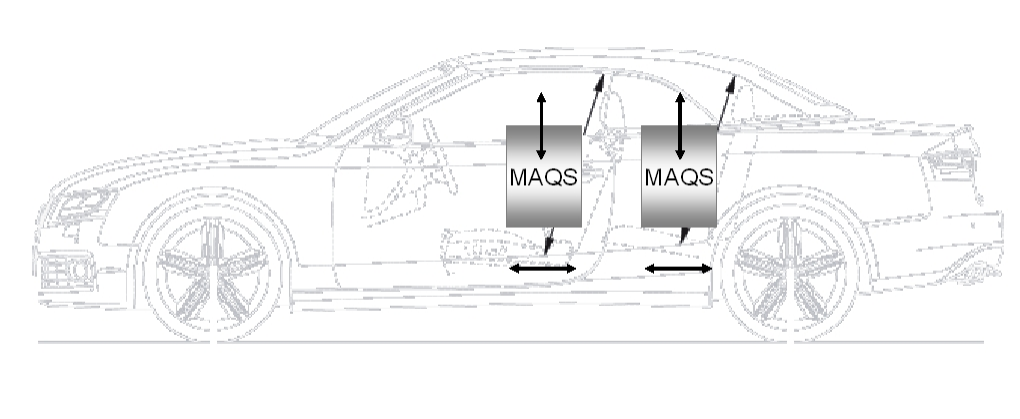
**Schematic illustration of MAQS sensing module**. The module consists of a supply unit and an analysis unit. In the analysis unit, particulate matter analyzer, gas analyzer (i.e. CO2, NO2, CO) and temperature, humidity, anemometer sensors are placed. An ultramobile PC unit integrates the data.

As primary technology platform, convertible vehicles will be used. In different vehicle types, the MAQS sensing modules will be placed. They consist of a supply unit and an analysis unit. In the analysis unit, particulate matter analyzers, gas analyzers (i.e. CO2, NO2, CO) and temperature, humidity, anemometer etc. sensors are placed (fig. 2). An ultramobile PC unit integrates the data.

In a second step, bicycle, motor bicycle and pedestrian solutions will be developed.

Monitoring will be carried out using convertible vehicles in open and closed positions. Driving conditions will be standardised to represent typical urban behaviours for the different seasons of the year. Vehicles will be driven with either open or closed windows and convertible tops, with air-conditioning turned on or off and with varied settings of the ventilation system. Prior to the first analysis run on each route, the vehicles will be ventilated for at least 5 minutes with open doors. The routes will be chosen in different settings: "Traffic zone", motor way or suburban and other urban and rural settings including tunnels. Depending on traffic conditions, these will be analysed and categorized differently. Data will be averaged from replicates in order to provide estimates of exposure for a distinct situation. Also, timing of the analysis routes (which may include pre-defined intermediate waypoints or randomized routes) will be monitored by electronic watches which are synchronised against the different monitoring devices and GPS-systems. Also, wind speed will be measured once on each route, using anemometers (detection limit 0.1 m/s). The data can also be compared to meteorologic and emission outdoor air parameters. Referring to this, the Berlin Luftgütemessnetz (BLUME) may be used for studies in the German capital Berlin. The data is presented online by the Senatsverwaltung Berlin [5] and will be analysed and compared to the data recorded in the vehicle. Data analysis and comparison will be performed using a specifically computed software that integrates the vehicle analysis system measurements with the BLUME measurements [5].

In the vehicle, the analysers will be located on the back seats to simulate the weakest passenger possible in a car: a child. Other locations will be co-driver seats. Averaging time for measurements will range between 1 and 60 seconds, depending on the target parameter.

### Public Access

A major target of MAQS is to provide public access to the measurements. Ideally, MAQS will be used to establish mobile sensing systems on a nation-wide and European scale. It may be used by governmental and non-governmental institutions for information. The analysed environmental exposure data will be connected to GPS data and presented in the internet.

## Discussion

So far, mobile air pollutant analysing system on the basis of convertible vehicles did not reach large scale practical implementation. Therefore, only little data is available in public databases such as PubMed. Previously, a number of studies have used particulate matter analysis in closed vehicles. In this respect, two studies assessed the exposure to fine airborne particulate matter (PM_2.5_) in closed vehicles [6,7]. It was reported that this may be associated with cardiovascular events and increased mortality in older and cardiac patients. Potential physiologic effects of in-vehicle, roadside, and ambient PM_2.5 _were investigated in young, healthy, nonsmoking, male North Carolina Highway Patrol troopers [7]. Nine troopers (age 23 to 30) were monitored on 4 subsequent days while working a 3 P.M. to midnight shift. Each patrol car was equipped with air-quality monitors. Blood was drawn 14 hours after each shift, and ambulatory monitors recorded the electrocardiogram throughout the shift and until the next morning [7]. Data were analysed using mixed models. In-vehicle PM_2.5 _(average of 24 μg/m^3^) was associated with decreased lymphocytes (-11% per 10 μg/m^3^) and increased red blood cell indices (1% mean corpuscular volume), neutrophils (6%), C-reactive protein (32%), von Willebrand factor (12%), next-morning heart beat cycle length (6%), next-morning heart rate variability parameters, and ectopic beats throughout the recording (20%) [7]. Controlling for potential confounders had little impact on the measured effects. The correlations of these health endpoints with ambient and roadside PM_2.5 _were smaller and less significant. The measurements in these healthy young men suggested that in-vehicle exposure to PM_2.5 _may cause pathophysiological changes including inflammation, coagulation and cardiac rhythm changes [7].

Another study assessed particulate matter concentrations whilst simultaneously walking and driving 48 routes in London, UK [8]. Car trips were performed with closed windows and the moderate ventilation system settings. It was shown that mean exposures while walking were greatly in excess of those while driving, by a factor 4.7 for the coarse particle mass (PM10-PM2.5), 2.2 for the fine particle mass (PM2.5-PM1), 1.9 for the very fine particle mass (<PM1) and 1.4 for ultrafine particle number density [8]. It is enticing to speculate how convertible vehicle measurements would have been. With the ability of the MAQS-platform, this analysis can be performed in future. The reduced in-car exposures was attributed to the filtration system which helped to prevent ingress of particles, so that the vehicle acted as a more-or-less independent micro-environment, insulated against much of air pollution present in the street [8].

In contrast to results from closed vehicles, exposure in open vehicles has not been investigated in great detail so far. In this respect, the present project may not only be used as mobile traffic pollution sensor platform but also to investigate the particulate matter exposure in open-convertible vehicles versus closed-convertible vehicles under a multitude of settings.

Concerning other mobile environmental sensing systems, a recent British project may be used as a benchmark. This project entitled Mobile Environmental Sensing System Across Grid Environments (MESSAGE) is a three-year research project that is funded jointly by the British Engineering and Physical Sciences Research Council and the British Department for Transport [9]. Besides this, MESSAGE also has the support of nineteen non-academic organisations from public sector transport operations, commercial equipment providers, systems integrators and technology suppliers [9].

Beginning in October 2006, nearly 4 million EURO are invested to develop and demonstrate the potential of diverse, low cost sensors and to provide data for the planning, management and control of the environmental impacts of transport activity at urban, regional and national level in the United Kingdom. As in the MAQS-project which focuses on Germany, MESSAGE includes the implementation on vehicles. In addition, pedestrians are recruited to act as mobile, real-time environmental sensor carriers in order to sense transport and non-transport related pollutants and hazards [9].

Within the project, three sensor platforms are developed: The University of Cambridge group investigates the potential for mobile phones to support a sensing system. The University of Newcastle develops a "smart-dust" network using Zigbee (IEEE 802.15.4) motes, and the Imperial College in London devises a network that utilises WiFi (IEEE 802.11.g) and WiMax (IEEE 802.16) technologies for communications and positioning [9].

With this British project as benchmark, the MAQS-platform is intended to provide a first mobile environmental sensing system for Germany using convertible vehicles as a new technology platform. Information and updates on MAQS are available on the internet portal of the Institute of Occupational Medicine of the Charité [10].

## Competing interests

The authors declare that they have no competing interests.

## Authors' contributions

DAG, CS, AF, DQ, BK conceived of the study, and participated in its design and coordination. ML, MT, TCF, SK, AvM, SU, UW, KV, FB, SM, CK, BK, AF, HZ, AG, JB, KAM, JD, MVG, CP, PW, RJ, HB, MG, MS, JPA, JAB, A-MJ, SG, AS, NN, SK, SH, LK, SM, CP, UH, RR, US, EK, GW, AM-F, HH, AdR, ST, MW, JM, JS, AS, SF, CS, AB, ON, ND, QTD, HG, BK, RNBG, CW, SW, LZ, JS, BK, ECM, NS, VU, DQ are project partners and participate in the conductance of the study. All authors read and approved the final manuscript.
